# Developing as a Physician: Visual Arts in Medical Education

**DOI:** 10.1212/NE9.0000000000200151

**Published:** 2024-09-05

**Authors:** Emily Schwartz, Dominique Harz, Brooke DiGiovanni Evans

**Affiliations:** From the Harvard Medical School (E.S., D.H.); and Department of Medicine (B.D.E.), Brigham & Women's Hospital, Boston, MA.

As we approached the doorway, I (E.R.S.) stopped—suddenly mindful of the present moment. These were the final seconds of serenity for the couple sitting on the other side of the threshold. I wanted to wait—gift them the bliss of normalcy for a few seconds longer in my pause. But, I followed as the neurologists entered the room. The resident began the conversation, explaining the finding of a malignant brain tumor on the MRI and its devastating implications to the couple before us. As he spoke, I found myself taking in the unfolding events from 2 discrete perspectives. As a medical student, I was impressed by the resident—his calm, clarity, compassion, and composure as he led the conversation were remarkable. As a daughter, I was profoundly heartbroken—I saw my parents in the couple's faces as I relived the moment we learned of my father's advanced cancer diagnosis.

As a second-year medical student, I feel as though I hold 2 distinct identities. One, I inhabited before entering medical school; the second, I have just begun to inhabit as a medical student. This duality highlights the process of professional identity formation, a lifelong process that integrates cognitive, social, and emotional capacities. While essential to medical education, this journey demands the cultivation of skills such as empathy, reflective practice, and a deeper understanding of the human condition. Guiding students through this transformation can be challenging. The visual arts, with their unique characteristics, offer a promising avenue to support and enhance this complex process of identity formation in medical students.

Art can bridge the gap between personal and professional identities, emphasizing the humanity inherent in medical practice and offering a space to reflect both individually and as a group.^1^ This is exemplified by a novel Arts and Humanities workshop at the Harvard Art Museum that I participated in as a first-year medical student. Using visual arts–based methodologies (Table), the workshop required no previous art experience and provided a forum for us medical students to reflect on our experiences both before beginning medical school and during the first year of our training. This workshop helped shape our professional identities, enhancing our understanding and empathy as future physicians.

**Table:** Art-Based Activities Used in the Workshop

Art-based activity	Description	Value in medical education
Visual Thinking Strategies (VTSs)	Developed by Abigail Housen and Philip Yenawine, VTS involves a discussion led by a trained facilitator focused on building a group interpretation through responses to 3 questions: What is going on in this work of art? What do you see that makes you say that? What more can you find?^2^	VTS demonstrates the importance of multiple perspectives and the role of visual evidence and teamwork in developing an interpretive analysis. It is a valuable methodology that can also be used as a reflective exercise to promote critical thinking, clinical diagnosis, and observation skills.^4-7^
Personal Responses Tour	Developed by Ray Williams, a museum educator, the Personal Responses Tour asks students to reflect on an individual question prompt and find a work of art that connects to that prompt and has a personal resonance.^8^	Personal Responses Tour provides a psychologically safe space for self-reflection and open discussion about a variety of topics and emotions. This activity can help strengthen community and shared understanding among participants.
Drawing activity	The drawing activity was based on research by Dr. Helen Riess on empathy.^12^ In this exercise, students pair up to draw each other's eyes using the blind contour drawing technique (drawing without looking at the paper).	Drawing offers another learning modality to experience reflection. This activity encouraged students to slow down and make careful observations, demonstrating the power of eye contact and observation in the doctor-patient relationship.

The workshop began with visual thinking strategies (VTSs), a pedagogical method centered around the group exploration of artwork through a facilitated discussion.^2^ My classmates and I gathered around a painting by Gustave Moreau^3^ (Figure) and, without having been told the title of the piece, were instructed to silently observe. I personally focused on components of the background, parts of the image that the artist perhaps did not intend for us to notice first—the contrast of the small red birds against dark shadows and the faint yet striking eye that was looming over the sleeping child. When the instructor asked us about our observations, one by one people expressed what struck them. Each detail mentioned was discrete, reflecting where each person's eyes and mind were led. We all explored the artwork together—finding meaning, pondering different possibilities, and gaining a deeper understanding of the piece. One person, for example, believed that the painting was a rendering of Moses drifting in a basket down the Nile River. He was well versed in the biblical story and able to bring together certain symbolic imagery that others did not have the context to reference. As soon as this perspective was brought to light, those discrete details came together to form an alternative narrative, a new possibility.

**Figure:**
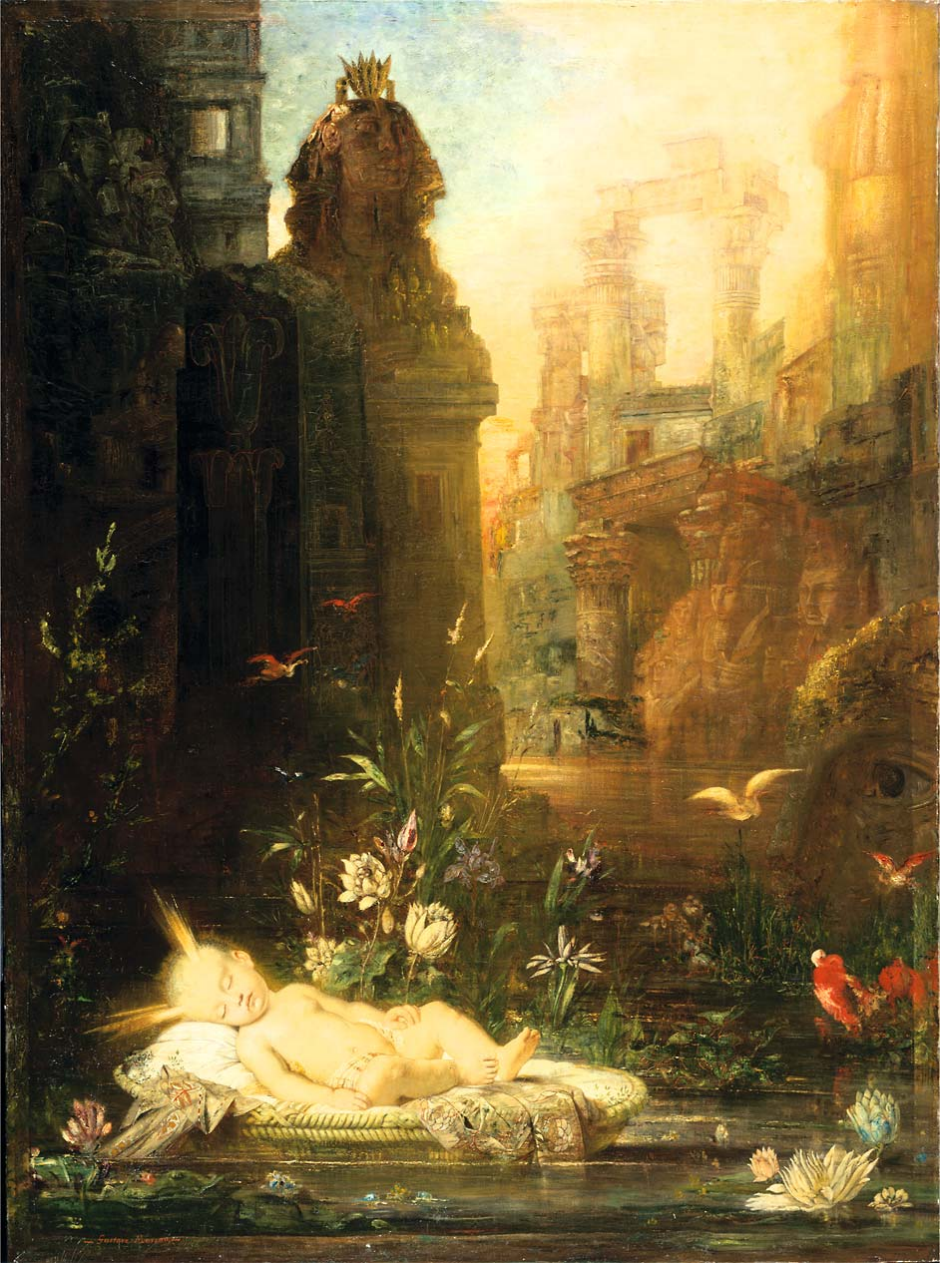
The Infant Moses by Gustave Moreau Reproduced with permission from Harvard Art Museums/Fogg Museum, Bequest of Grenville L. Winthrop, Photo © President and Fellows of Harvard College, 1943.262.

This exercise demonstrates the importance of incorporating a breadth of different viewpoints when drawing conclusions, so as to ensure a comprehensive understanding of a particular circumstance. With expert colleague collaboration, a healer must simultaneously integrate discrete data points of medical evidence and understand the patient and their family's illness experience. VTS highlights the value of shared perspective and the importance of heterogeneous observational contribution, as shown in multiple publications.^4-6^ Thus, VTS is a valuable tool that can be incorporated in clinical diagnosis courses and even be used for bedside teaching.^7^

The second part of the workshop, Personal Responses Tour,^8,9^ prompted us to select a work of art and draw a connection to 1 of 3 themes—collaboration, flourishing, or empathy. When the empathy prompt instructed me to “find a work of art that you'd share with a friend who concerns you,”^8^ my instinct was to try to find a piece that would create a safe launching point from which I could engage a friend I was concerned about in deep conversation. I stopped at a collection of medallions, each of which depicted a different element of suffering. These medallions reminded me of similar religious medals that my grandfather held close, and I remembered how he endured many of the aspects of suffering portrayed by the art before me. I stood in silence, pondering how the human experience is unified by the anguish rendered in these depictions, humbled by the depths of the moments we must endure to reap the reward of cherishing life. When my classmates and I were brought back together to share our reflections, I was not expecting what so beautifully unfolded. One of my peers stood by a sculpture that I had walked by and most likely would not have stopped to take in had she not brought it to our attention. In the asymmetrical cubes connected to each other by rods of various widths, she saw her grandmother's kitchen, she smelled her food, and suddenly this sculpture transformed before my eyes. I was there alongside her, experiencing her childhood memory.

The Personal Responses Tour is an effective activity to promote reflection and build community.^9-11^ The prompts used in this workshop encouraged students to reflect on patient-centered care, emphasizing shared humanity. Patients are too commonly reduced to a case or a diagnosis in clinical practice. This activity was a reminder to never forget each patient's narrative and the importance of appreciative inquiry. As physicians, being curious is fundamental in understanding the ways a patient's illness takes shape in their life. Taking the time to listen to how one's health holds value for them is imperative in providing ethical, compassionate, and empathetic health care.^10^

A drawing activity, based on Dr. Helen Riess' work on empathy, concluded the workshop. Dr. Riess explains the need to focus on people's eyes and faces as one of the tools to build empathic capacity.^12^ We formed pairs and sketched one another's eyes using the blind contour drawing technique—drawing without looking at the paper—allowing us to focus on looking without judging our drawing. I looked into my partner's eyes and was immediately taken aback by the intimacy. Humbled by the authenticity of the moment, I focused solely on the task at hand—drawing the eyes without lifting my pencil off the paper. Then suddenly, he broke out in laughter. It was so intense that he needed a break. I felt it too. A relief.

This last activity was a compelling representation of a fundamental, but sometimes forgotten, aspect of the clinical encounter: the importance of truly seeing our patients, looking into their eyes. Furthermore, it fostered reflection on the reciprocal nature of observation in the doctor-patient relationship. Just as doctors observe and examine, patients observe their doctors—reading their faces for concern, interpreting body language, sensing the haste when there is too much to be done in too little time, and detecting hesitation when the words are hard to find. As such, we, as doctors, are being observed as much as we embody the role of the observer.

This workshop demonstrated the ways in which art can be a bridge between the discrete professional and experiential identities, which allows them to merge into the self, so that both worlds coexist in humble service. Deepening medical students' reflection on the human experience—as inspired by the visual arts—can foster the development of students' professional identity and also strengthen their collective identity as health care providers who commit to humanistic and ethical patient care.^1^ Furthermore, by engaging with the arts, students can cultivate essential skills such as empathy, reflective practice, tolerance to ambiguity, resilience, flexibility, teamwork, communication, and a deeper understanding of the human condition.^1^ This aligns with the personal and professional development (PPD) competencies, which underscore the necessity for training programs to assess, teach, and guide learners in developing these skills. Incorporating art-based methodologies into medical training can play a crucial role in guiding students toward mastering these (PPD) competencies, which are vital for their growth as competent and compassionate professionals.^13^

Throughout the course of my clinical training, I have been growing in my ability to hold space for my past and am learning how to keep such experiences present at the patient's bedside. The love and longing that I will always hold for my father and the pride and heartbreak that I feel for my mother will contribute to my ability to honor the illness experience of another. As I left the workshop, the patient and his wife were at the forefront of my mind. I felt a strengthened responsibility to communicate to each patient for whom I care that the meaning that they ascribe to their circumstance is not only understood but valued by their medical team.

## Appendix Authors

**Table:**

Name	Location	Contribution
Emily Schwartz	Harvard Medical School, Boston, MA	Drafting/revision of the manuscript for content, including medical writing for content
Dominique Harz	Harvard Medical School, Boston, MA	Drafting/revision of the manuscript for content, including medical writing for content
Brooke DiGiovanni Evans	Department of Medicine, Brigham & Women's Hospital, Boston, MA	Drafting/revision of the manuscript for content, including medical writing for content

## References

[1] Howley L, Gaufberg E, King BE. The Fundamental Role of the Arts and Humanities in Medical Education. Association of American Medical Colleges; 2020.

[2] Yenawine P. Visual Thinking Strategies: Using Art to Deepen Learning Across School Disciplines. Harvard Education Press; 2013.

[3] Moreau G. The Infant Moses. In: Harvard Art Museums/Fogg Museum, Bequest of Grenville L. Winthrop, Photo © President and Fellows of Harvard College, 1943.262.

[4] Katz JT, Khoshbin S. Can visual arts training improve physician performance? Trans Am Clin Climatol Assoc. 2014;125:331-342. 25125749 PMC4112699

[5] Chisolm MS, Kelly-Hedrick M, Wright SM. How visual arts–based education can promote clinical excellence. Acad Med. 2021;96(8):1100-1104. doi:10.1097/ACM.0000000000003862 33264111

[6] Strohbehn GW, Hoffman SJ, Tokaz M, et al. Visual arts in the clinical clerkship: a pilot cluster-randomized, controlled trial. BMC Med Educ. 2020;20:481. doi:10.1186/s12909-020-02386-w 33256727 PMC7708096

[7] Miller A, Grohe M, Khoshbin S, Katz JT. From the galleries to the clinic: applying art museum lessons to patient care. J Med Hum. 2013;34(4):433-438. doi:10.1007/s10912-013-9250-8 24014232

[8] Williams R. Honoring the personal response: a strategy for serving the public hunger for connection. J Mus Educ. 2010;35(1):93-102. doi:10.1080/10598650.2010.11510653

[9] Gaufberg E, Williams R. Reflection in a museum setting: the personal responses tour. J Grad Med Educ. 2011;3(4):546-549. doi:10.4300/JGME-D-11-00036.1 23205206 PMC3244323

[10] Chisolm M, Kelly-Hedrick M, Stephens M, Zahra F. Transformative learning in the art museum: a methods review. Fam Med. 2020;52(10):736-740. doi:10.22454/FamMed.2020.622085 33151534

[11] Gaufberg E, Batalden M. The third thing in medical education. Clin Teach. 2007;4(2):78-81. doi:10.1111/j.1743-498x.2007.00151.x

[12] Riess H, Neporent L. The Empathy Effect: Seven Neuroscience-Based Keys for Transforming the Way We Live, Love, Work, and Connect Across Differences. Sounds True; 2018.

[13] Englander R, Cameron T, Ballard AJ, Dodge J, Bull J, Aschenbrener CA. Toward a common taxonomy of competency domains for the health professions and competencies for physicians. Acad Med. 2013;88(8):1088-1094. doi:10.1097/ACM.0b013e31829a3b2b 23807109

